# Kentucky Lunatic Asylum

**Published:** 1846-03

**Authors:** 


### THE WESTERN JOURNAL.

New Series.—Vol. V.—No. III.

LOUISVILLE, MARCH 1, 1846.

---

### KENTUCKY LUNATIC ASYLUM.

We believe it is admitted, on all hands, that the Lunatic Asylum, at Lexington, does not meet the wants of the community, and is deficient in many things necessary to the comfort and successful treatment of the insane. These defects are pointed out in two communications to the Legislature, which have been placed on our table;—one the "Annual Report of the Directors and Physician;" the other a "Memorial, by Miss D. L. Dix, soliciting an appropriation" for the asylum, "and also urging the necessity for establishing a new hospital in the Green river country." We fervently hope that this prayer may be granted, and that the funds solicited with reference to extending the buildings, and augmenting the means of rendering the patients comfortable, will be placed in the hands of the board of directors without delay. It would be unworthy of legislators in this enlightened age to hesitate about such an appropriation when such interests are at stake. The asylum can never become what such an institution ought to be; especially, it can never produce the delightful results now everywhere looked for from retreats for the insane—the recovery of a large proportion of recent cases of insanity, —without substantial changes and improvements, to effect which the institution must have funds. Kentucky is not satisfied with her lunatic asylum. It does not hold out sufficient inducements to the friends of deranged persons to send them early to the hospital, and thus the period most favorable to a cure is allowed to pass away. Owing to this cause, and others enumerated in the report of the physician, our asylum has not kept pace with other similar institutions, which, from their signal success in restoring the insane to reason, are estemeed among the chief glories of our age.

We heartily approve of the policy of establishing in Kentucky another asylum for lunatics, but it occurs to us that the site for it is Louisville. Not only is Louisville the point most accessible from all parts of Kentucky, but its position on the river would attract the insane who were able to pay from the States of Indiana, Illinois, Arkansas, and Mississippi. Indeed, as several of those States have yet no lunatic hospital, it is probable that their legislatures might be induced to unite with Kentucky in the establishment, at Louisville, of an asylum which should be open to all. In this way, States which may not be able for many years to erect their own hospitals, would have at once a retreat for this unfortunate class of their population. At Louisville the best professional skill could be secured, while its constant and quick communication with all parts of the Mississippi Valley, would afford opportunities for sending the insane to the hospital at an early stage of their disease, a circumstance of the utmost importance since the chances of cure, it is well understood, diminish with the duration of the insanity.

The public are waking up to a sense of their obligations to this class of the afflicted. We are beginning to feel, that lunatics have claims upon our sympathies, and ought to be treated with gentleness and kindness—not locked up, like criminals, in common jails, or dreary houses built expressly for their confinement, to swelter in filth, and be devoured by vermin. But what is done with them where there are not retreats in which they may be made secure, and provided with the comforts of life? The answer "would a tale unfold" too disgusting for the ears of polite society.

